# Influence of Physicians

**Published:** 1855-03

**Authors:** 


INFLUENCE OF PHYSICIANS.—Of the list of persons who were recently elected to the Board of School Committee in Boston, which consists of seventy-two, nearly one-fifth of them were physicians.

